# Factors Associated with IgG/IgM Levels after SARS-CoV-2 Vaccination in Patients with Head and Neck Cancer

**DOI:** 10.3390/tropicalmed9100234

**Published:** 2024-10-08

**Authors:** Wei Liao, Haoyu Liang, Yujian Liang, Xianlu Gao, Guichan Liao, Shaohang Cai, Lili Liu, Shuwei Chen

**Affiliations:** 1 State Key Laboratory of Oncology in South China, Guangdong Provincial Clinical Research Center for Cancer, Collaborative Innovation Center for Cancer Medicine, Guangzhou 510060, China; liaowei@sysucc.org.cn (W.L.); lianghy@sysucc.org.cn (H.L.); gaoxl@sysucc.org.cn (X.G.); liulil@sysucc.org.cn (L.L.); 2 Department of Intensive Care Unit, Sun Yat-sen University Cancer Center, Guangzhou 510060, China; 3 Department of Pathology, Sun Yat-sen University Cancer Center, Guangzhou 510060, China; 4 Department of Pediatrics, Sun Yat-sen University First Affiliated Hospital, Guangzhou 510060, China; lyujian@mail.sysu.edu.cn; 5 Department of Head and Neck Surgery, Sun Yat-sen University Cancer Center, Guangzhou 510060, China; 6 Department of Infectious Diseases, Nanfang Hospital, Southern Medical University, Guangzhou 510515, China; liaoguichan2023@gmail.com

**Keywords:** immunochemotherapy, IgG/IgM, head and neck cancer, SARS-CoV-2 vaccination, COVID-19

## Abstract

This study evaluated the factors influencing IgG/IgM antibody levels in 120 patients with head and neck cancer (HNC) following vaccination with inactivated SARS-CoV-2 vaccines. Each patient’s demographic and clinical data were documented, and serum IgG and IgM antibodies were detected using a commercial magnetic chemiluminescence enzyme immunoassay kit. The results indicated that while all patients had received at least one vaccine dose, 95 tested positive for IgG and 25 were negative. A higher proportion of IgG-positive patients had received three vaccine doses. Comparatively, gamma-glutamyl transferase levels were elevated in IgM-negative patients. The study further differentiated patients based on their treatment status: 46 were treatment-naive and 74 had received chemotherapy combined with immune checkpoint inhibitors (ICT) at enrollment. Despite similar baseline characteristics and time from vaccination to antibody detection, IgM positivity was significantly lower in the ICT group, with no significant difference in IgG positivity between the treatment-naive and ICT groups. A multivariable analysis identified the number of vaccine doses as an independent factor of IgG positivity, while ICT emerged as an independent risk factor for IgM positivity. Additionally, IgG titers generally declined over time, although patients with higher baseline IgG levels maintained higher titers longer. In conclusion, ICT in patients with HNC does not significantly affect IgG levels post-vaccination. However, booster vaccinations have been shown to be associated with higher IgG positivity, although these levels gradually decrease over time.

## 1. Introduction

Although the zenith of the COVID-19 outbreak may have receded, isolated cases persisted. Besides wearing personal protective equipment in high-risk settings, COVID-19 vaccination remains pivotal in averting infection or reinfection with SARS-CoV-2 [1,2,3]. Numerous studies have confirmed the capacity of COVID-19 vaccines to decrease new infections and mitigate severe cases, ultimately reducing mortality rates [4,5,6,7]. These beneficial effects are particularly evident in immunocompromised patient cohorts, including those with HIV or cancer [4,5,6].

While the COVID-19 pandemic has subsided, sporadic cases continue to emerge, underscoring the need for timely vaccination, particularly among immunocompromised populations. Understanding how these vulnerable groups, especially cancer patients, respond to vaccination efforts is crucial due to their increased risk for severe COVID-19-related morbidity and mortality. Studies have shown that cancer patients infected with SARS-CoV-2 experience prolonged viral shedding, delayed seroconversion, and T-cell phenotypic exhaustion [7]. Those with lung cancer, hematologic malignancies, and patients receiving stem cell transplants or adoptive cellular therapies face the highest risk [8,9]. Effective measures, including SARS-CoV-2 vaccination, have been implemented to protect these individuals. However, the exclusion of cancer patients and other immunocompromised groups from COVID-19 vaccine trial registrations necessitates that vaccine effectiveness can only be elucidated through small-scale observational studies focusing on immune or antibody responses [10].

Head and neck cancer (HNC) is one of the most prevalent malignancies in the world. According to Global Cancer Statistics, HNC is the seventh most commonly diagnosed cancer, with 890,000 new cases reported solely in 2020 [11,12,13]. Its prevalence varies across regions and populations. Prognosis depends on various factors, including the disease stage, tumor size, lymph node involvement, distant metastases, and overall health status of patients [14,15]. Cancer patients are a vulnerable group for SARS-CoV-2 infection, making it crucial to analyze the factors related to their antibody production after vaccination.

More importantly, the varying responses to vaccines demand ongoing research to optimize vaccination strategies, ensuring robust immune protection for all, especially those at higher risk due to compromised immune systems. Currently, various types of COVID-19 vaccines, including inactivated vaccines, have been developed and deployed worldwide, with the inactivated vaccines being the most prevalent in China. Given the high incidence of head and neck cancers in this region, it is imperative to analyze how inactivated vaccines perform specifically in this patient group. The effectiveness of these vaccines is typically assessed through the detection of immune responses, specifically the presence of immunoglobulins such as IgM and IgG. Given the vulnerability of cancer patients, research may focus more on the effects of antibody production rather than solely on clinical outcomes like virological breakthroughs, symptomatic COVID-19, or COVID-19-related hospitalizations and deaths.

The merging of immunotherapy targeting immune checkpoints with neoadjuvant chemotherapy holds promise as a paradigm-shifting HNC treatment, substantially boosting survival rates substantially [16,17,18]. Nonetheless, this immunochemotherapy (ICT) approach may unwittingly trigger undesirable effects, such as viral reactivation, marrow suppression, and immune-related organ impairment [19,20,21,22]. Moreover, whether ICT affects the efficacy of the SARS-CoV-2 vaccine in HNC patients remains an unanswered question. It raises concerns about the interplay between the treatment of fundamental diseases and vaccine-induced antibody titers.

Numerous studies have assessed vaccine effectiveness in patients with tumors, yet there remains an insufficient focus on the humoral response induced by vaccines during anti-cancer treatment. The interaction between immunotherapy combined with chemotherapy (ICT) and vaccination is particularly underexplored. Previous reports suggest that ICT might influence viral activation [23,24,25]. It raises questions about its impact on the efficacy of inactivated vaccines. Despite the potential significance of these interactions, data specifically addressing the effects of ICT on the response to inactivated vaccines in cancer patients is still limited. This gap in the research highlights the urgent need for targeted studies to elucidate the implications of ICT on inactivated vaccine-induced immunity.

Hence, we embarked on an observational cross-sectional study by enrolling HNC patients with HNC who had undergone at least one shot of an inactivated SARS-CoV-2 vaccination. We evaluated the levels of IgG and IgM produced after vaccination and analyzed the factors related to antibodies. Our primary objective was to evaluate the influencers, including treatment options, on the SARS-CoV-2 vaccine response in this specific cohort

## 2. Subjects and Methods

### 2.1. Patient Enrollment

**Figure 1:**
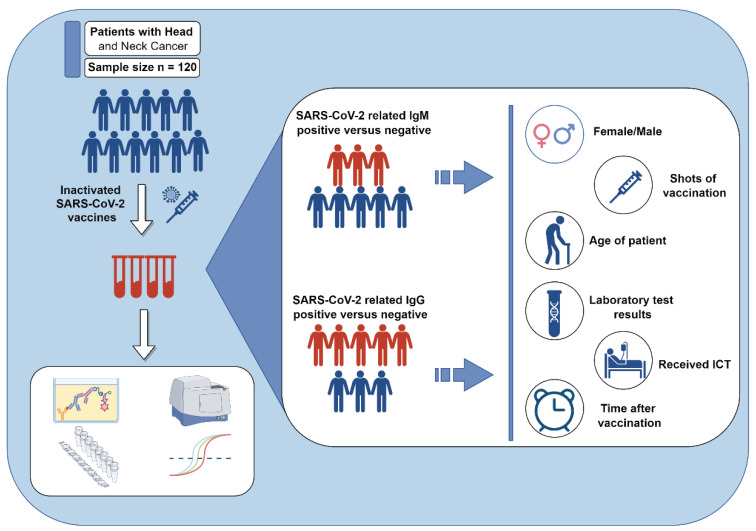
**A schematic figure of the study.** This observational cross-sectional study involved 120 patients with head and neck cancer. All patients had received at least one shot of inactivated SARS-CoV-2 vaccine. We aim to evaluate the factors influencing IgG/IgM antibody levels in those patients following vaccination with inactivated SARS-CoV-2 vaccines. This Figure was created by Figdraw (www.figdraw.com, accessed on 14 September 2024).

A schematic figure is shown in Figure 1. This observational cross-sectional study involved 120 patients with head and neck cancer (HNC), each diagnosed based on verified pathological reports. All participants had received at least one shot of inactivated SARS-CoV-2 vaccine. Comprehensive data, including demographics, biochemical markers, and blood cell counts, were collected and recorded. Exclusion criteria included symptoms of recent upper respiratory infections, missing critical data such as SARS-CoV-2 infection history, recent plasma treatments, and pregnancy. Patients were also excluded if they were diagnosed with a secondary tumor, such as neurogenic cancer. To confirm the absence of current SARS-CoV-2 infection, nasopharyngeal swab specimens were collected from all patients at enrollment. All patients enrolled in this study received inactivated SARS-CoV-2 vaccines. Of the patients, 46 received the Sinopharm COVID-19 vaccine, 35 received the CoronaVac COVID-19 vaccine, and 39 received a combination of both vaccines above. All patients enrolled were confirmed to have no prior SARS-CoV-2 infection before specimen collection. The study was approved by the Ethics Committee of Sun Yat-sen University Cancer Hospital, and informed consent was obtained from all participants.

### 2.2. Immunochemotherapy and Laboratory Analysis

In this study, during the collection of specimens for IgG/IgM testing, the patient group was divided based on their treatment status: those who had already received ICT treatment were classified as the ICT group, while those yet to receive ICT treatment were classified as the control group. Routine blood cell counts were estimated using Sysmex XN9000 (Sysmex Corporation, Kobe, Japan). Serum biochemical parameters, including alanine aminotransferase (ALT) and albumin (ALB) levels, were estimated using the Roche Cobas 702 (Roche Diagnostics GmbH, Mannheim, Germany). These evaluations were performed in accordance with the manufacturer’s guidelines.

### 2.3. Detection of SARS-CoV-2-Specific IgG and IgM

In our study, all patients enrolled received inactivated vaccines. The average interval between the final vaccination dose and the blood sampling for IgM/IgG testing was 7.75 ± 3.49 months. Serum samples from all patients were inactivated before the assessment of IgG and IgM levels against the SARS-CoV-2 RBD protein. All samples were inactivated at 56 °C for 30 min. The magnetic chemiluminescence enzyme immunoassay kit (Vazyme, DD3112-P-01, and DD3111-01, Vazyme Medical Technology Co, Nanjing, China) strictly followed the operating steps described in the user manual.

IgG Detection Using Indirect ELISA Method: The kit detects RBD IgG antibodies against SARS-CoV-2 through a two-step incubation process. Initially, the microwells on the enzyme-labeled plate are coated with recombinant RBD proteins. When SARS-CoV-2 RBD IgG antibodies are present in the sample, they bind to this pre-coated antigen. The process involves washing the wells to remove unbound substances, then adding enzyme-labeled secondary antibodies that specifically bind to human IgG. After another wash, a color development solution is added and the plate is incubated away from light. Finally, the absorbance is read at a wavelength of 450 nm, with the intensity indicating the quantity of IgG antibodies. Specific Steps for IgG detection: Sample Addition, Incubation, Washing, Enzyme Label Addition, Incubation, Repeat Washing, Color Development, and Reading the absorbance at 450 nm. The IgM Detection Steps are listed as followed: Dilution, Sample Addition to Plate (add 100 μL of diluted standard or sample to an anti-human IgM protein-coated plate), Incubation at 37 °C for 1 h, then wash three times with washing solution, add 100 μL of diluted SARS-CoV-2 (BA.4) Spike (S) Protein RBD (His-Tag) (HRP Conjugated), and incubate at 37 °C for 45 min, then wash four times, Color Development and Reading the absorbance at 450 nm. The threshold levels used to assess IgG/IgM positivity or negativity are aligned with the manufacturer’s recommendations.

### 2.4. Statistical Analysis

Normally distributed measurements were expressed as mean ± standard deviation (SD). Categorical data were presented as percentages. Comparative analyses were performed using t-tests and one-way ANOVA when appropriate. Pearson’s correlation, receiver operating characteristic (ROC) curve calculations, and logistic regression were used. SPSS software (version 13.0) was used, with significance set at 0.05.

## 3. Results

### 3.1. Clinical Variables Related to IgM against SARS-CoV-2 Titers

We next examined clinical indicators associated with IgM titers. The results are shown in Supplementary Table S1. A total of 19 patients tested with positive IgM while 101 patients tested negative. We found that, between IgM-negative and IgM-positive patients, the gamma-glutamyl transferase levels were significantly higher in IgM-negative patients (*p* = 0.002). Gamma-glutamyl transferase is a glycoprotein bound to the cell membrane. When hepatocytes sustain damage, gamma-glutamyl transferase is released into the bloodstream. Clinically, it is widely utilized as a diagnostic marker for detecting liver function abnormalities.

### 3.2. Clinical Variables Related to IgG against SARS-CoV-2 Titers

Among the patients enrolled, 95 patients tested positive for IgG against SARS-CoV-2, while 25 tested negatives. We explored the variables that influenced the IgG positivity rate. As shown in Supplementary Table S2. The IgG positivity rate was associated with the number of shots in SARS-CoV-2 vaccinations.

**Figure 2:**
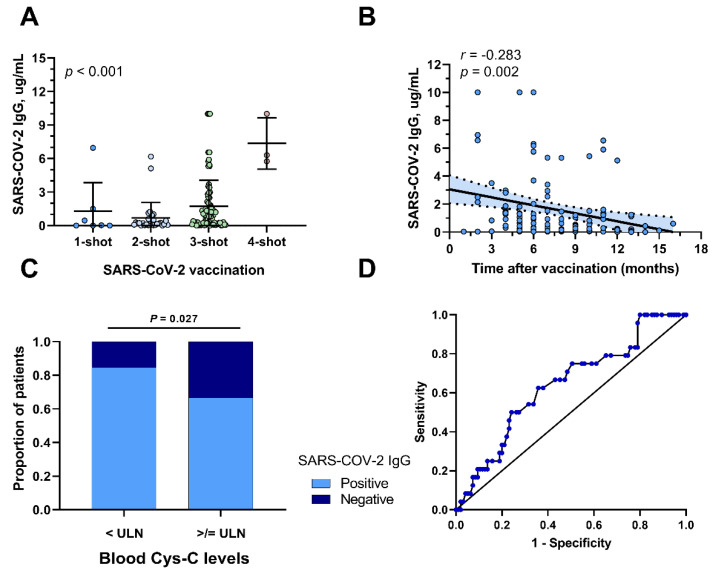
**Clinical Variables Associated with IgG Titers.** (**A**). IgG titers for patients receiving 1-shot, 2-shot, 3-shot, and 4-shot vaccinations were 1.28 ± 2.56, 0.69 ± 1.39, 1.73 ± 2.33, and 7.36 ± 2.31, respectively (*p* < 0.001). (**B**). There was a significant negative correlation (r = −0.28, *p* = 0.002) between IgG titer and time after vaccination. (**C**). The IgG-positive rate was 84.9% for patients with normal cystatin C and 66.7% for those with elevated cystatin C (*p* = 0.027). (**D**). The area under the curve for cystatin C level predicting IgG positivity was 0.64 (95%CI 0.52–0.76, *p* = 0.035). ULN: Upper Limit of Normal.

We further analyzed the variables associated with IgG titers. We found that IgG titers were also correlated positively with the number of shots of SARS-CoV-2 vaccination (*p* < 0.001, Figure 2A) while IgG titers were negatively correlated with the time after vaccination (r = −0.283, *p* = 0.002, Figure 2B). In our study, we also found a notable association between cystatin C (Cys-C) levels and the SARS-CoV-2 IgG positivity rate (Figure 2C). Specifically, HNC patients with elevated blood Cys-C levels demonstrated a correspondingly reduced IgG positivity rate compared with other patients (*p* = 0.027). Cys-C serves as an endogenous marker of a glomerular filtration rate, accurately reflecting changes in renal filtration. It is recognized as a highly specific and reliable method for the early detection of renal function impairment, making it an ideal indicator for such evaluations. ROC analysis corroborated the predictive prowess of Cys-C in determining IgG positivity, with an area under the curve of 0.64 (95%CI 0.52–0.76, *p* = 0.035, Figure 2D).

**Figure 3:**
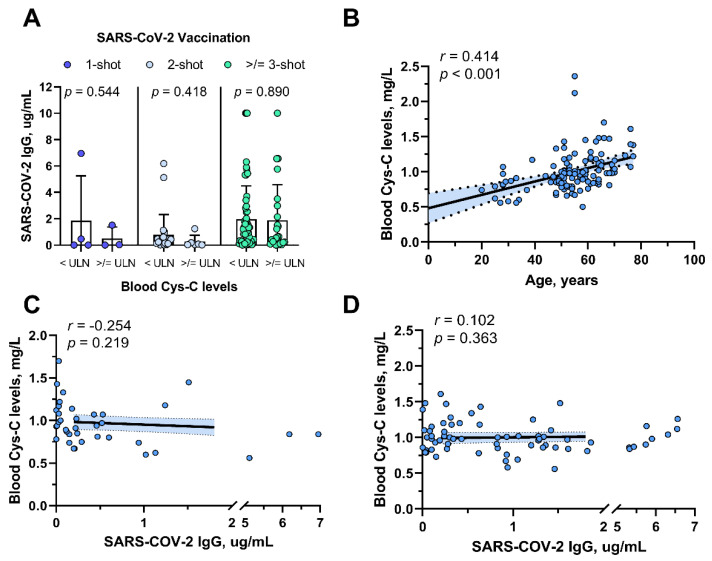
**Association between cystatin C levels and IgG Titers.** (**A**). Among patients receiving one shot, two shots, and more than three shots of vaccine, IgG titers for patients with normal cystatin C and elevated cystatin C were 1.85 ± 3.41 vs. 0.51 ± 0.86 ug/mL (*p* = 0.544), 0.79 ± 1.54 vs. 0.26 ± 0.48 ug/mL (*p* = 0.418), and 1.98 ± 2.52 vs. 1.89 ± 2.69 (*p* = 0.890), respectively. (**B**). Cystatin C level was significantly positively correlated with age (r = 0.362, *p* = 0.001). (**C**). No significant correlation was observed between Cystatin C and IgG levels in patients who received 1–2 shots of vaccination. (**D**). No significant correlation was observed between Cystatin C and IgG levels in patients who received 3–4 shots of vaccination. ULN: Upper Limit of Normal.

Patients with impaired renal function seems to have a poorer vaccine response. However, the influence of Cys-C on IgG titers diminishes with an increasing number of vaccination shots (Figure 3A). Additionally, we observed a significant positive correlation between the Cys-C levels and patient age (r = 0.414, *p* < 0.001; Figure 3B). However, we did not observe a significant negative correlation between the Cys-C levels and IgG antibodies in patients, regardless of whether they received 1–2 or 3–4 shots of the vaccine, as shown in Figure 3C,D. Notably, there seems to be a trend indicating that as the number of vaccine shots increases, the correlation between Cys-C and IgG becomes increasingly less relevant.

**Figure 4:**
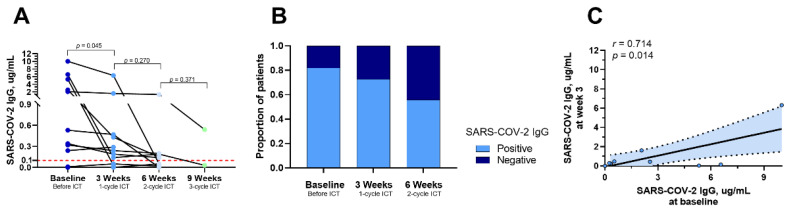
**Dynamic Changes of IgG Titers in HNC Patients after Receiving ICT.** (**A**). IgG titers for HNC patients before receiving ICT and after 1 cycle, 2 cycles, and 3 cycles of ICT were 2.54 ± 3.34, 0.89 ± 1.85, 0.23 ± 0.42, and 0.28 ± 0.36 ug/mL. (**B**). IgG-positive rates before ICT and after 1 cycle and 2 cycles of ICT were 81.8%, 72.7%, and 55.6%, respectively. (**C**). IgG titer before ICT was significant;y positively correlated with IgG titer after one cycle of ICT (r = 0.714, *p* = 0.014).

Eleven patients underwent continuous sampling both before and after ICT to evaluate dynamic changes in IgG titers. We observed a gradual decline in the IgG titers over time (Figure 4A). Correspondingly, the positivity rate of IgG showed a steady decrease over time (Figure 4B). Furthermore, a trend observed in patients with higher baseline IgG titers subsequently displayed higher IgG titers despite declining IgG titers over time (r = 0.714, *p* = 0.014, Figure 4C).

### 3.3. Impact of ICT on SARS-CoV-2 Antibody Titers

To further evaluate the factors associated with antibody levels after vaccination, we analyzed the impact of ICT on antibody titers after vaccination. A total of 46 patients enrolled were treatment-naïve, while others had already received chemotherapy combined with immune checkpoint inhibitors (*n* = 74) when enrolled. The baseline characteristics were well matched between the two groups (Supplementary Table S3), and the vaccination intervals (time after vaccination for SARS-CoV-2 IgG/IgM detection) were comparable (*p* = 0.575).

**Figure 5:**
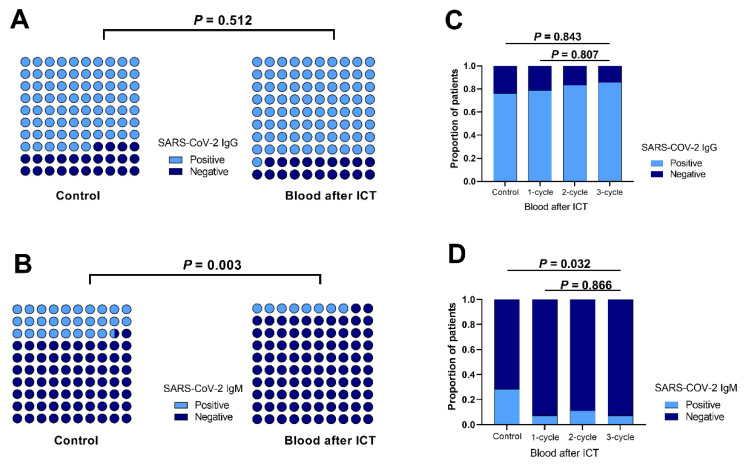
**Impact of Immunochemotherapy on IgG/IgM positivity rate.** (**A**). The IgG-positive rate in the control group (35/46) was 76.1%, and 60/74 (81.1%) in ICT group (*p* = 0.512). (**B**). The IgM-positive rate in the control group was 13/46 (28.3%), significantly higher than 6/74 (8.1%) in ICT group (*p* = 0.003). (**C**). IgG-positive rate of patients in control group was 76.1% and in patients in the ICT group who had received one, two, and three cycles ICT were 78.6%, 83.3%, and 85.7%, respectively (*p* = 0.843). (**D**). For the ICT group, IgM-positive rates for patients in control group was 28.3%, while in patients who had received one cycle, two cycles, and three cycles of ICT were 7.1%, 11.1%, and 7.1%, respectively (*p* = 0.032).

We noticed, as shown in Figure 5, that the positive IgG rates did not significantly differ between the treatment-naïve group and ICT groups. Conversely, IgM positivity was lower in the ICT group (*p* = 0.003), as shown in Figure 5A,B. Subgroup analyses within the ICT group revealed that the number of ICT cycles did not influence the IgG-positive rate (Figure 5C). Although there was a significant difference in the IgM positivity rate between patients who received ICT and those who did not, no significant difference was observed in the IgM positivity rate among patients who received ICT, regardless of the number of ICT cycles received (Figure 5D).

**Figure 6:**
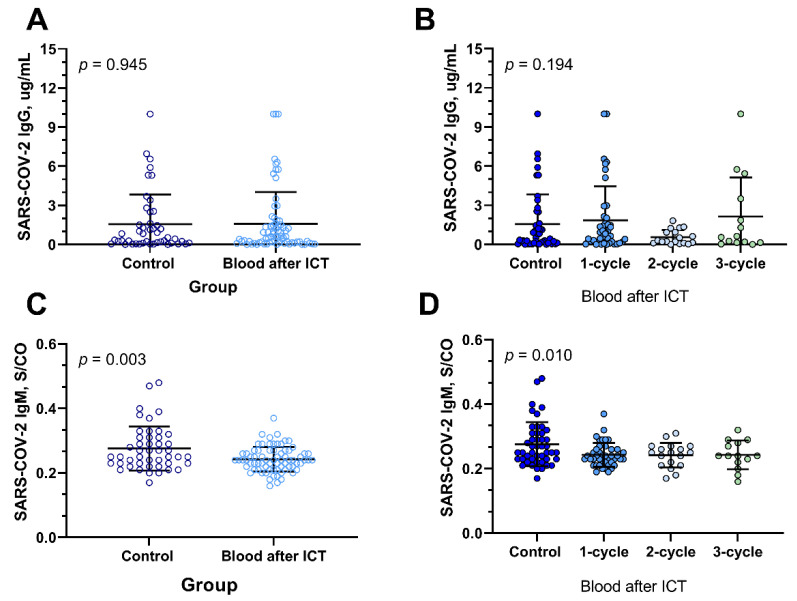
**Impact of Immunochemotherapy on IgG/IgM titers.** (**A**). The IgG titer in control group was 1.56 ± 2.28 ug/mL, compared with 1.59 ± 2.43 in ICT group (*p* = 0.945). (**B**). IgG titers in control group and in ICT patients who had received one cycle, two cycles, and three cycles of ICT were 1.56 ± 2.28, 1.86 ± 2.62, 0.56 ± 0.56, and 2.13 ± 3.01, respectively (*p* = 0.194). (**C**). The IgM titer in the control group was 0.28 ± 0.07, and for the ICT group, it was 0.24 ± 0.04 (*p* = 0.003). (**D**). IgM titers in the control group and ICT patients who received one cycle, two cycles, and three cycles of ICT were 0.28 ± 0.07, 0.24 ± 0.03, 0.24 ± 0.04, and 0.24 ± 0.04, respectively (*p* = 0.010).

Further analysis reveals that ICT did not affect IgG titers (Figure 6A,B). However, compared to the control group without ICT, a notable reduction in IgM titers was observed in the ICT group (Figure 2C,D).

### 3.4. Factors Associated with SARS-CoV-2 IgG/IgM Positivity

**Figure 7:**
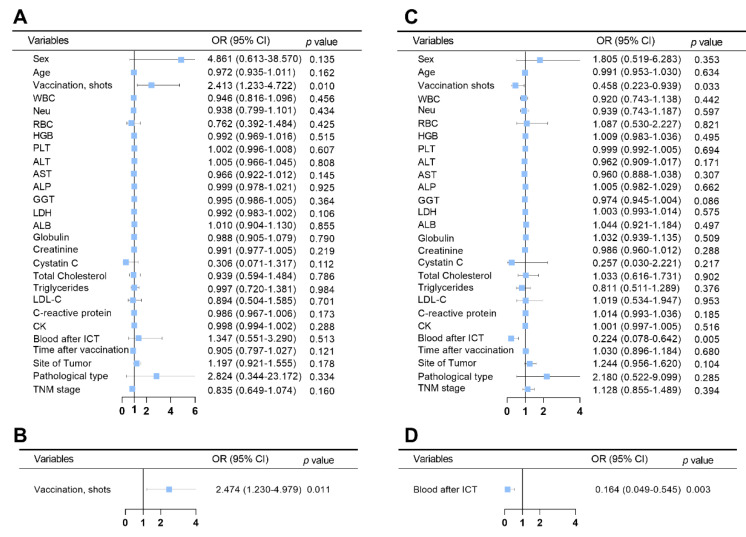
**Univariate and Multivariate Analysis of IgG/IgM positivity after Vaccination in HNC Patients.** (**A**). Univariate analysis of IgG positivity in HNC patients after SARS-CoV-2 vaccination. (**B**). Multivariate analysis of IgG positivity in HNC patients after SARS-CoV-2 vaccination. (**C**). Univariate analysis of IgM positivity in HNC patients after SARS-CoV-2 vaccination. (**D**). Multivariate analysis of IgM positivity in HNC patients after SARS-CoV-2 vaccination.

Univariate analyses revealed that the number of vaccination shots was associated with IgG positivity in HNC patients. The results were confirmed by multivariate analysis, which highlighted that the number of vaccinations was an independent factor associated with IgG positivity (OR = 2.474, *p* = 0.011) (Figure 7A,B).

A univariate analysis indicated that the number of shots in vaccination and ICT was associated with IgM positivity in patients with HNC (Figure 7C). A multivariate analysis showed that ICT was an independent risk factor for IgM positivity in patients with HNC (OR = 0.164, *p* = 0.003) (Figure 7D).

## 4. Discussion

In this study, we found that inactivated vaccines effectively induced humoral responses in HNC patients. Notably, receiving a booster vaccine was associated with positive anti-SARS-CoV-2 IgG in HNC patients, while ICT treatment was linked to negative anti-SARS-CoV-2 IgM. ICT administration did not significantly impact the vaccine-induced IgG positivity rate or titer. Furthermore, regardless of the number of ICT cycles received, no significant changes in the IgG positivity rates or titers were observed among the HNC patients. Our findings highlight that the number of vaccine shots is an independent factor for SARS-CoV-2 IgG positivity in patients with HNC. Our study also raises concerns about relying solely on IgM for COVID-19 diagnostic guidance, as it could lead to missed diagnoses, particularly in HNC patients with SARS-CoV-2 infection who have undergone ICT and, consequently, have decreased IgM titers

The prognosis for recurrent or metastatic HNC remains challenging. Before the introduction of immunotherapy, cetuximab, a monoclonal antibody targeting the epidermal growth factor receptor (EGFR), was the latest therapeutic agent added to the treatment options [26]. However, cetuximab has shown only a modest benefit, extending survival by an average of 2.7 months compared to platinum-based chemotherapy [27]. Recently, the treatment landscape for HNC has been significantly transformed by the advent of immune checkpoint inhibitors. These agents have become essential components of therapeutic strategies, particularly for managing recurrent or metastatic head and neck squamous cell carcinoma [28]. Pembrolizumab, the first anti-PD-1 therapy approved by the FDA for this indication, demonstrated promising efficacy in the KEYNOTE-012 study and was further validated in the phase III KEYNOTE-040 trial [28,29]. Similarly, nivolumab was approved based on its performance in the CheckMate 141 trial, which marked a significant shift in the management of this cancer [30]. These developments highlight how immunotherapy has revolutionized the treatment paradigm for head and neck cancer, offering new hope and improved outcomes for patients with this disease. However, the effect of immune checkpoint inhibitors on the immunogenicity of inactivated SARS-CoV-2 vaccines remains underexplored and is a focal point of our study.

IgM antibodies, structured in pentameric or hexametric forms, play a pivotal role in the early immune response [31]. At the onset of B-cell ontogeny, IgM is the first antibody class produced upon exposure to an antigen. While IgM antibodies generally exhibit a lower antigen-binding affinity than IgG, their high avidity allows them to bind non-protein antigens, such as carbohydrates and lipid antigen [31,32,33]. In our study, we found that IgM positivity and titers were lower in the ICT group than those in the control group. A similar result was found in a previous study, which showed that chemotherapy decreased the mean IgM level in patients [34]. Similar results were observed in this study. The ICT-induced IgM decline in our study may be due to chemotherapy rather than immunotherapy targeting the immune checkpoints. The underlying reasons for the decline in vaccination-induced IgM titers among cancer patients undergoing chemotherapy remain unclear. However, this raises an additional concern: whether this phenomenon might increase the risk of misdiagnosis. This prompts the question of whether solely relying on IgM for diagnostic guidance instead of nasopharyngeal swabs for viral nucleic acid detection could potentially lead to missed diagnoses of COVID-19. This scenario may gain significance, particularly in the context of patients with HNC who have undergone ICT and have been infected with SARS-CoV-2.

The research indicates a parallel kinetic pattern of IgM and IgG following SARS-CoV-2 infection, although IgM titers seem to decay faster [35,36,37,38]. A previous study showed that IgM is typically detectable around day 7 post-infection, with a decline starting at around day 27 [39]. In our study, the average time from vaccination to antibody detection was approximately eight months, potentially explaining the relatively low IgM positivity rate. Furthermore, our study confirmed a gradual decline in IgG titers over time, consistent with the natural course of immunity and independent of ICT treatment.

In our investigation, we observed that levels of gamma-glutamyl transferase were significantly elevated in patients who tested negative for IgM antibodies. While the specific mechanisms underlying this association remain unclear, existing research indicates that individuals with liver disease, particularly chronic conditions, may exhibit compromised immune responses, resulting in an incomplete protective effect from vaccination [40]. Additionally, studies have identified high gamma-glutamyl transferase levels at admission as independent predictors of ICU hospitalization and mortality among COVID-19 patients [41]. Gamma-glutamyl transferase plays a critical role in glutathione metabolism and the regulation of the redox balance [42]. This may be one of the possible reasons for its association with the COVID-19 inactivated vaccine response. However, our multivariate correlation analysis did not establish gamma-glutamyl transferase as an independent factor associated with IgM levels; rather, it highlighted that ICT was independently associated with IgM levels. This suggests that while gamma-glutamyl transferase levels correlate with IgM negativity, they do not directly influence IgM antibody responses independently of other factors. Given these findings, further research is warranted to elucidate the relationship between gamma-glutamyl transferase levels and IgM antibody responses in HNC patients following an inactivated vaccine

Our findings reveal an association between renal function and SARS-CoV-2-related IgG following vaccination. Specifically, we observed that higher levels of cystatin C, indicative of impaired renal function, were associated with a decreased likelihood of positive IgG post-vaccination. This pattern may highlight the potential need for booster doses in patients with compromised renal function to attain adequate immunogenicity. This observation is consistent with existing research, which suggests that deteriorating renal function may reduce vaccine efficacy [43,44]. Consequently, our study underscores the critical importance of booster vaccinations for this vulnerable population to ensure robust immune protection.

In our study, we observed a positive correlation between the number of vaccinations and elevated IgG titers, corroborating the findings of a previous study [45]. Likewise, a negative association between the post-vaccination time and IgG titers was confirmed [46,47]. In addition, we established a link between Cys-C levels and IgG-positivity rates. Cys-C serves as a marker for kidney function [48,49]. We also found that Cys-C levels positively correlated with patient age. Patients with chronic kidney disease are known to exhibit reduced vaccine efficacy, attributed to premature immune aging and chronic low-grade inflammation [50,51]. Intriguingly, our subgroup analysis indicated that although HNC patients with higher Cys-C levels displayed lower SARS-CoV-2 IgG positivity rates, increasing the number of vaccination shots in patients with higher Cys-C yielded IgG titers similar to those in patients with lower Cys-C levels. This finding suggests that a sufficient number of vaccination shots may be helpful for immune response in this subgroup.

The objective of SARS-CoV-2 vaccination is to elicit sustained humoral responses and generate effective neutralizing antibodies that confer protection against SARS-CoV-2 infection. However, the dynamic monitoring of IgG titers in an 11-patient subset revealed that IgG titers gradually declined over time. Thus, patients with HNC may remain susceptible to COVID-19 even after a certain period of vaccination. While ICT did not appear to influence IgG positivity or titers, we observed no abnormal increase in IgG levels post-ICT treatment in these patients. It is reported that immunotherapies targeting immune checkpoints can activate specific viruses [52,53]. Given that none of the patients in our study experienced SARS-CoV-2 infection by the end of the study period, the impact of immunotherapy on individuals with a history of SARS-CoV-2 infection remains uncertain.

Our study had some limitations. First, this was a single-center study with a relatively small sample size. Moreover, the follow-up duration in this prospective cohort was relatively short. Although the ICT-treated HNC patients in our study received the SARS-CoV-2 vaccination, whether the infection rate is different from that of patients who did not receive ICT after exposure to the real world awaits further research.

## 5. Conclusions

In our study exploring the effect of inactivated SARS-CoV-2 vaccines among HNC patients and the impact of ICT on vaccine-induced immunity, we uncovered that while ICT does not alter IgG positivity rates or titers, it is associated with reduced IgM levels post-vaccination. Our multivariate analysis further emphasizes the importance of booster vaccinations as an independent factor associated with SARS-CoV-2 IgG positivity in these patients. These insights highlight the need for tailored vaccination schedules, including timely and potentially additional booster doses, to optimize COVID-19 protection for HNC patients, thereby informing more effective clinical management strategies in this vulnerable population.

## Data Availability

The authors confirm that all relevant data are included in the article, and the materials are available upon reasonable request from the authors and the data was deposited in the Research Data Deposit repository (NO. RDDA2024591716).

## Supplementary Materials

The following supporting information can be downloaded at: https://www.mdpi.com/article/10.3390/tropicalmed9100234/s1, Supplemental Table S1. Characteristics of patient with IgM negative or positive after vaccination. Supplemental Table S2. Characteristics of patient with IgG negative or positive after vaccination. Supplemental Table S3. Characteristics of patient had received ICT or treatment naïve.

## Abbreviations

ICT: immunochemotherapy; HNC, head and neck cancer; ALT, alanine aminotransferase; ALB, albumin; S/CO, signal-to-cutoff; ROC, receiver operating characteristic; Cys-C, cystatin C.
