# Supplementary material for: Factors Associated with IgG/IgM Levels after SARS-CoV-2 Vaccination in Patients with Head and Neck Cancer
Source: Trop Med Infect Dis. 2024 Oct 8;9(10):234. doi: 10.3390/tropicalmed9100234 (PMC11511189; doi:10.3390/tropicalmed9100234)
Supplement: Supplementary file 1 [file tropicalmed-09-00234-s001.zip › tropicalmed-3178584-supplementary.pdf]

Table S1. Characteristics of patient who are IgM-negative or -positive after vaccination.

Baseline Variable	SARS-Cov-2 antibody detection in HNC patients		*P* value
	IgM-Negative	IgM-Positive	
**Sample Size**	101	19	-
**Sex, M/F**	88/13	15/4	0.348
**Age, years**	54.57 ± 12.29	53.11 ± 13.08	0.637
**SARS-CoV-2 Vaccine**			0.150
1 shot	5 (5.0%)	2 (10.5%)	
2 shots	22 (21.8%)	8 (42.1%)	
3 shots	71 (70.3%)	9 (47.4%)	
4 shots	3 (3.0%)	0 (0%)	
**TNM stage**			0.180
Stage I-II	14 (13.9%)	3 (15.8%)	
Stage III-IV	68 (67.3%)	9 (47.4%)	
Uncertain	19 (18.8%)	7 (36.8%)	
**Tumor site**			0.381
oral cavity	31 (30.7%)	4 (21.1%)	
oropharyngeal	18 (17.8%)	3 (15.8%)	
larynx	18 (17.8%)	3 (15.8%)	
hypopharynx	17 (16.8%)	2 (10.5%)	
Others	17 (16.9%)	7 (36.8%)	
**Pathological type, SCC/NSCC**	93/8	16/3	0.275
**Time after vaccination, months**	7.69 ±3.42	8.05 ±3.98	0.683
**White blood cells, ×10^9/L**	7.79 ±2.89	7.25 ±2.24	0.445
**Neutrophils, ×10^9/L**	5.26 ±2.55	4.93 ±2.26	0.599
**Red blood cells, , ×10^{12}/L**	4.54 ±0.69	4.58 ±0.66	0.822
**Hemoglobin, g/L**	133.18 ±19.98	136.53 ±17.95	0.498
**Platelets, ×10^9/L**	270.33 ±78.24	262.84 ±67.21	0.697
**Alanine aminotransferase, U/L**	20.71 ±12.31	16.66 ±8.24	0.172
**Aspartate aminotransferase, U/L**	20.09 ±9.05	17.90 ±4.69	0.306
**Alkaline phosphatase, U/L**	83.88 ±20.37	86.35 ±27.77	0.665
**Gamma-glutamyl transferase, U/L**	45.78 ±44.26	27.42 ±14.72	0.002
**Lactate dehydrogenase, U/L**	182.34 ±46.44	188.83 ±38.47	0.578
**Albumin, g/L**	44.52 ±3.95	45.21 ±4.45	0.500
**Globulin, g/L**	28.63 ±5.33	29.52 ±3.93	0.512
**Serum creatinine, umol/L**	79.17 ±29.94	71.97 ±20.68	0.319
**Cystatin C**	1.01 ±0.29	0.92 ±0.25	0.219
**Total cholesterol**	4.87 ±0.99	4.90 ±1.07	0.903
**Triglycerides**	1.97 ±1.49	1.64 ±0.85	0.375
**Low-density lipoprotein**	3.03 ±0.80	3.04 ±0.81	0.954
**C reactive protein, mg/L**	9.42 ±17.54	16.06 ±26.97	0.172
**Creatine kinase, U/L**	106.23 ±107.29	123.95 ±115.62	0.516

TNM stage, Tumor-Node-Metastasis stage. Normal laboratory values are as follows: White blood cells: 4-10 x 10^9/L; neutrophils: 2-7.5 x 10^9/L; red blood cells: 4.0-5.50 x 10^12/L. Hemoglobin levels are 120-160 g/L for

males and 110-150 g/L for females. Platelets: 100-300 x 10^9/L. The upper limits of normal for alanine aminotransferase and aspartate aminotransferase are 40 U/L each. Alkaline phosphatase: 110 U/L; gamma-glutamyl transferase: 61 U/L; lactate dehydrogenase: 300 U/L; albumin: 35-55 g/L; globulin: 20-30 g/L. Serum Creatinine levels are 57-97 μmol/L. Cystatin C: 0.59-1.03 mg/L; triglycerides: 1.7 mmol/L; low-density lipoprotein: 3.10 mmol/L; creatine kinase: 310 U/L.

Table S2. Characteristics of patient with IgG-negative or -positive results after vaccination.

Baseline Variable	SARS-Cov-2 antibody detection in HNC patients		*P* value
	IgG-Negative	IgG-Positive	
**Sample Size**	25	95	-
**Sex, M/F**	24/1	79/16	0.101
**Age, years**	57.44 ± 11.56	53.53 ± 12.51	0.161
**SARS-CoV-2 Vaccine**			0.046
1 shot	4 (16.0%)	3 (3.2%)	
2 shots	8 (32.0%)	22 (23.2%)	
3 shots	13 (52.0%)	67 (70.5%)	
4 shots	0	3 (3.2%)	
**TNM stage**			0.489
Stage I-II	2 (8.0%)	15 (15.8%)	
Stage III-IV	16 (64.0%)	61 (64.2%)	
Uncertain	7 (28.0%)	19 (20.0%)	
**Tumor site**			0.256
oral cavity	9 (36.0%)	26 (27.4%)	
oropharyngeal	3 (12.0%)	18 (18.9%)	
larynx	7 (28.0%)	14 (14.7%)	
hypopharynx	4 (16.0%)	15 (15.8%)	
Others	2 (8.0%)	22 (23.2%)	
**Pathological type, SCC/NSCC**	24/1	85/10	0.314
**Time after vaccination, months**	8.72 ± 3.95	7.49 ± 3.34	0.119
**White blood cells, ×10^9/L**	8.08 ± 2.87	7.61 ± 2.78	0.456
**Neutrophils, ×10^9/L**	5.56 ± 2.49	5.11 ± 2.51	0.430
**Red blood cells, , ×10^{12}/L**	4.65 ± 0.54	4.53 ± 0.72	0.428
**Hemoglobin, g/L**	136.04 ± 17.98	133.13 ± 20.07	0.518
**Platelets, ×10^9/L**	262.00 ± 70.62	270.94 ± 78.01	0.611
**Alanine aminotransferase, U/L**	19.54 ± 8.38	20.19 ± 12.57	0.810
**Aspartate aminotransferase, U/L**	22.09 ± 11.68	19.15 ± 7.49	0.132
**Alkaline phosphatase, U/L**	84.63 ± 13.56	84.16 ± 23.27	0.926
**Gamma-glutamyl transferase, U/L**	50.08 ± 38.68	41.06 ± 42.33	0.357
**Lactate dehydrogenase, U/L**	197.33 ± 53.01	179.86 ± 42.63	0.097
**Albumin, g/L**	44.50 ± 3.30	44.67 ± 4.19	0.857
**Globulin, g/L**	29.02 ± 5.03	28.70 ± 5.18	0.792
**Serum creatinine, umol/L**	85.07 ± 18.54	76.23 ± 30.57	0.179
**Cystatin C**	1.08 ± 0.25	0.97 ± 0.29	0.101

**Total cholesterol**	4.93 ± 1.32	4.86 ± 0.91	0.789
**Triglycerides**	1.93 ± 1.24	1.92 ± 1.46	0.984
**Low-density lipoprotein**	3.08 ± 1.01	3.01 ± 0.75	0.704
**C reactive protein, mg/L**	15.45 ± 24.66	9.22 ± 17.72	0.160
**Creatine kinase, U/L**	130.46 ±115.01	103.65 ± 106.56	0.281

TNM stage, Tumor-Node-Metastasis stage. Normal laboratory values are as follows: White blood cells: 4-10 x 10^9/L; neutrophils: 2-7.5 x 10^9/L; red blood cells: 4.0-5.50 x 10^12/L. Hemoglobin levels are 120-160 g/L for males and 110-150 g/L for females. Platelets: 100-300 x 10^9/L. The upper limits of normal for alanine aminotransferase and aspartate aminotransferase are 40 U/L each. Alkaline phosphatase: 110 U/L; gamma-glutamyl transferase: 61 U/L; lactate dehydrogenase: 300 U/L; albumin: 35-55 g/L; globulin: 20-30 g/L. Serum Creatinine levels are 57-97 µmol/L. Cystatin C: 0.59-1.03 mg/L; triglycerides: 1.7 mmol/L; low-density lipoprotein: 3.10 mmol/L; creatine kinase: 310 U/L.

Table S3. Characteristics of patient who had received ICT or were treatment naïve.

Baseline Variable	HNC patients		*P* value
	Treatment-Naïve	Received ICT	
**Sample Size**	46	74	-
**Sex, M/F**	34/12	69/5	0.003
**Age, years**	53.91 ± 13.71	54.61 ± 11.57	0.766
**SARS-CoV-2 Vaccine**			0.251
1 shot	3 (6.5%)	4 (5.4%)	
2 shots	16 (34.8%)	14 (18.9%)	
3 shots	26 (56.5%)	54 (73.0%)	
4 shots	1 (2.2%)	2 (2.7%)	
**TNM stage**			0.007
Stage I-II	12 (26.1%)	5 (6.8%)	
Stage III-IV	23 (50.0%)	54 (73.0%)	
Uncertain	11 (23.9%)	15 (20.2%)	
**Tumor site**			0.097
oral cavity	11 (23.9%)	24 (32.4%)	
oropharyngeal	6 (13.0%)	15 (20.3%)	
larynx	8 (17.4%)	13 (17.6%)	
hypopharynx	6 (13.0%)	13 (17.6%)	
Others	15 (32.6%)	9 (12.2%)	
**Pathological type, SCC/NSCC**	37/9	72/2	0.002
**Time after vaccination, months**	7.97 ± 3.73	7.61 ± 3.36	0.575
**White blood cells, ×10^9/L**	7.29 ± 1.83	7.97 ± 3.25	0.200
**Neutrophils, ×10^9/L**	4.79 ± 1.48	5.47 ± 2.95	0.154
**Red blood cells, , ×10^{12}/L**	4.70 ± 0.57	4.45 ± 0.74	0.056
**Hemoglobin, g/L**	140.02 ± 16.13	129.74 ± 20.68	0.005
**Platelets, ×10^9/L**	261.78 ± 78.74	273.77 ± 75.01	0.407

**Alanine aminotransferase, U/L**	19.25 ± 9.88	20.57 ± 12.93	0.556
**Aspartate aminotransferase, U/L**	19.59 ± 10.02	19.84 ± 7.50	0.877
**Alkaline phosphatase, U/L**	80.85 ± 22.72	86.72 ± 20.49	0.160
**Gamma-glutamyl transferase, U/L**	42.47 ± 50.71	43.28 ± 33.99	0.920
**Lactate dehydrogenase, U/L**	178.62 ± 39.45	186.51 ± 48.67	0.361
**Albumin, g/L**	45.42 ± 3.38	44.15 ± 4.32	0.097
**Globulin, g/L**	28.89 ± 4.89	28.67 ± 5.33	0.822
**Serum creatinine, umol/L**	74.05 ± 18.72	80.52 ± 33.39	0.233
**Cystatin C**	0.93 ± 0.23	1.04 ± 0.31	0.059
**Total cholesterol**	4.88 ± 0.99	4.87 ± 1.01	0.950
**Triglycerides**	1.69 ± 1.02	2.09 ± 1.63	0.154
**Low-density lipoprotein**	3.05 ± 0.78	3.01 ± 0.82	0.766
**C reactive protein, mg/L**	9.76 ± 18.63	10.93 ± 19.93	0.749
**Creatine kinase, U/L**	110.83 ± 97.45	107.95 ± 115.33	0.888

TNM stage, Tumor-Node-Metastasis stage. Normal laboratory values are as follows: White blood cells: 4-10 x 10^9/L; neutrophils: 2-7.5 x 10^9/L; red blood cells: 4.0-5.50 x 10^12/L. Hemoglobin levels are 120-160 g/L for males and 110-150 g/L for females. Platelets: 100-300 x 10^9/L. The upper limits of normal for alanine aminotransferase and aspartate aminotransferase are 40 U/L each. Alkaline phosphatase: 110 U/L; gamma-glutamyl transferase: 61 U/L; lactate dehydrogenase: 300 U/L; albumin: 35-55 g/L; globulin: 20-30 g/L. Serum Creatinine levels are 57-97 µmol/L. Cystatin C: 0.59-1.03 mg/L; triglycerides: 1.7 mmol/L; low-density lipoprotein: 3.10 mmol/L; creatine kinase: 310 U/L.
